# Rotary Structural Color Spindles from Droplet Confined Magnetic Self‐Assembly

**DOI:** 10.1002/advs.202207270

**Published:** 2023-01-17

**Authors:** Hanxu Chen, Shuangshuang Miao, Yuanjin Zhao, Zhiqiang Luo, Luoran Shang

**Affiliations:** ^1^ Department of Rheumatology and Immunology Nanjing Drum Tower Hospital School of Biological Science and Medical Engineering Southeast University Nanjing 210096 China; ^2^ Oujiang Laboratory (Zhejiang Lab for Regenerative Medicine, Vision and Brain Health) Wenzhou Institute University of Chinese Academy of Sciences Wenzhou Zhejiang 325001 China; ^3^ Shanghai Xuhui Central Hospital, Zhongshan‐Xuhui Hospital, and the Shanghai Key Laboratory of Medical Epigenetics International Co‐laboratory of Medical Epigenetics and Metabolism (Ministry of Science and Technology), Institutes of Biomedical Sciences Fudan University Shanghai 200032 China

**Keywords:** colloidal crystal, droplet, magnetism, self‐assembly, spindle stirrer, structural color

## Abstract

Structural colors materials are profoundly explored owing to their fantastic optical properties and widespread applications. Development of structural color materials bearing flexible morphologies and versatile functionalities is highly anticipated. Here, a droplet‐confined, magnetic‐induced self‐assembly strategy for generating rotary structural color spindles (SCSPs) by fast solvent extraction is proposed. The as‐prepared SCSPs exhibit an orderly close‐packed lattice structure, thus appearing brilliant structural colors that serve as encoding tags for multiplexed bioassays. Besides, benefitting from the abundant specific surface area, biomarkers can be labeled on the SCSPs with high efficiency for specific detection of analytes in clinical samples. Moreover, the directional magnetic moment arrangement enables contactless magnetic manipulation of the SCSPs, and the resultant rotary motions of the SCSPs generates turbulence in the detection solution, thus significantly improving the detection efficiency and shortening the detection time. Based on these merits, a portable point‐of‐care‐testing strip integrating the rotary SCSPs is further constructed and the capability and advantages of this platform for multiplexed detection of tumor‐related exosomes in clinical samples are demonstrated. This study offers a new way for the control of bottom‐up self‐assembly and extends the configuration and application values of colloidal crystal structural colors materials.

## Graphical abstract

Rotary structural color spindle is generated by a droplet‐confined, magnetic‐induced self‐assembly strategy via fast solvent extraction. A portable point‐of‐care‐testing strip integrating the rotary particles is constructed and demonstrated with the capability and advantages for multiplexed detection of tumor‐related exosomes in clinical samples. It is highly promising for the control of bottom‐up self‐assembly and extends the configuration and application values of colloidal crystal structural colors materials.

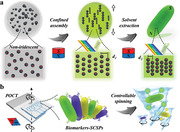

## 1 Introduction

Structural colors, derived from the interaction between light and materials with periodically arranged nanostructures bearing different reflective indices, have been widely identified in natural objects and living creatures, like opals, butterfly wings and feathers of certain birds.^[^ ^1^, ^2^, ^3^, ^4^, ^5^, ^6^, ^7^, ^8^ ^]^ Inspired by these natural phenomena, a series of strategies have been developed to produce artificial structural color materials by mimicking specific periodic architectures including template replication, 3D printing, and self‐assembly.^[^ ^9^, ^10^, ^11^, ^12^, ^13^, ^14^, ^15^, ^16^, ^17^ ^]^ Among these methods, self‐assembly provides a bottom‐up strategy for rapid and easy‐prototyping preparation of structural color materials. Particularly, confined assembly of colloidal nanoparticles in droplets result in the generation of colloidal crystal particles (CCPs).^[^ ^18^, ^19^, ^20^, ^21^, ^22^, ^23^ ^]^ The close‐packed structures of CCPs gives rise to iridescent structural color and the particulate morphology make them available as microcarriers for extensive applications, such as displaying, encoding, sensors, etc.^[^ ^24^, ^25^, ^26^, ^27^, ^28^, ^29^, ^30^, ^31^, ^32^, ^33^, ^34^, ^35^ ^]^ Although great progress has been achieved in droplet templated‐CCPs, their primary morphology is limited to be spherical due to the act of surface tension during assembly. This largely restricts their practical applications since the shape of particles matters for their performances, especially when motion, orientation, and particle interaction are involved.^[^ ^36^, ^37^, ^38^, ^39^ ^]^ Therefore, novel strategies for the fabrication of structural color particles possessing more flexible morphologies and great functionalities are still highly anticipated.

**Figure 1:**
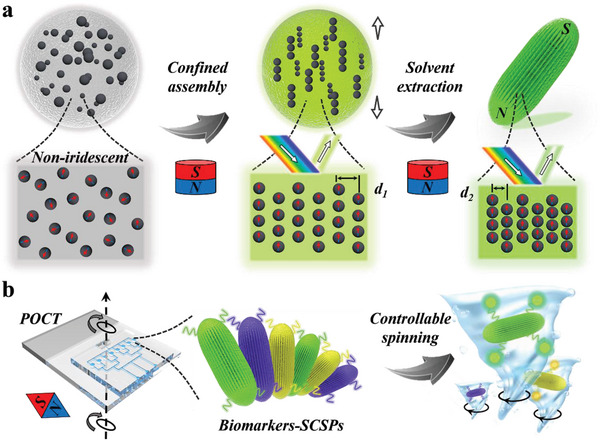
Schematic diagram of the fabrication process and clinical applications of the SCSPs. a) The magnetism‐induced, droplet‐confined self‐assembly strategy of the SCSPs. b) The SCSPs‐integrated POCT strip for tumor diagnosis with the assistance of rotary turbulence caused by controllable spinning.

In this paper, we developed a novel strategy for producing structural color particles with desired spindle morphology via magnetic self‐assembly, as schemed in **Figure** **1**a. Magnetism provides a powerful driving force for the assembly of nanoscale objects into certain spatial arrangement. During the assembly process, magnetic interactions can be combined with other effects, e.g., shape and curvature, thus offering delicate control over the geometry of the final product.^[^ ^40^, ^41^ ^]^ With that, smart materials with tailored superstructures including 1D magnetic chains/wires, 2D nanosheets, and 3D crystals have been well‐developed, and have found widespread applications in sensors, robotics, and bioanalysis.^[^ ^42^, ^43^, ^44^, ^45^ ^]^ Taking advantage of this, we herein conducted magnetism‐assisted assembly of magnetic colloidal nanoparticles within droplet templates. By controlling the magnetic field and the solvent extraction process, the nanoparticles assembled into a close‐packed periodic nanoarchitecture, while the droplet interface gradually deformed from spherical to spindle shape, finally generating structural color spindles (SCSPs). The unique spindle shape and the magneto‐responsiveness of the SCSPs enabled their use of as rotary stirrers, and the structural color served as distinguishable tags for encoding multiple probe molecules. Based on this, the SCSPs served as rotary spindle barcodes for multiplexed bioassays. Besides, we integrated the SCSPs into a point‐of‐care‐testing (POCT) strip for specific detection of tumor cells‐derived exosomes. We demonstrated that the turbulences induced by controllable spinning of the SCSPs was effective in improving the detection efficiency. These features indicated that the magnetic‐induced confined assembly strategy breaks the limitation of traditional assembly methods on the morphology of colloidal crystals. The resultant rotary SCSPs are ideal encoding barcodes for multiplex bioassay and the present POCT platform brings great opportunities for auxiliary clinical cancer diagnosis.

## 2 Results and Discussion

**Figure 2:**
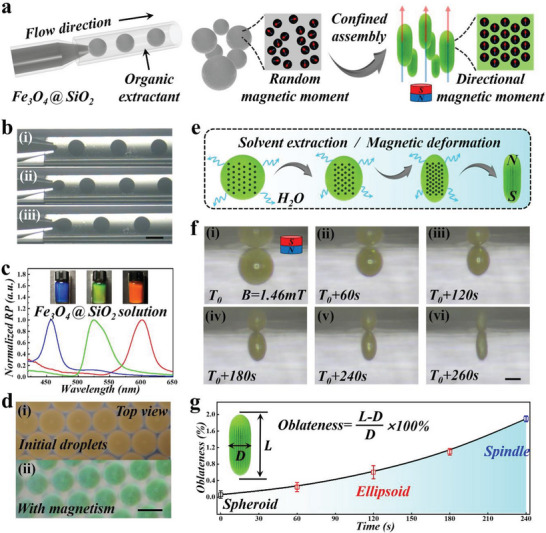
Characterization of the assembly and deformation process of the SCSPs. a) Schematic diagram of the magnetism‐mediated, droplet‐templated self‐assembly mechanism. b) Real‐time microscopy images showing the generation of the droplets in the microfluidic channel. c) Reflection spectra and corresponding optical images of the Fe_3_O_4_@SiO_2_ suspensions bearing blue, green, and orange structural colors under magnetic field. The nanoparticles assembled as close‐packing under magnetic field and appeared structural colors. d) Reflection images of droplets at i) the initial state and ii) magnetism‐induced state from top view. e) Schematic of the shape alteration from spheroid to spindle along with water extraction and magnetism‐induced stretching. f) Reflection images of a droplet at sequential time points from the initial state to the final spindle under constant external magnetic field. g) The definition of the oblateness of the SCSP and the oblateness variations with time. Scale bars are 500 µm in (b, d) and 150 µm in (f).

In a typical experiment, the SCSPs were fabricated based on magnetically‐induced droplet‐confined self‐assembly of core–shell magnetic colloidal nanoparticles (Fe_3_O_4_@SiO_2_) in microfluidic droplet templates, as shown in **Figure** **2**a. Monodispersed Fe_3_O_4_@SiO_2_ nanoparticles were synthesized via a modified Stöber method as reported before.^[^ ^46^ ^]^ To improve the stability and biocompatibility, a silica shell was coated on the surface of the Fe_3_O_4_ nanoparticles, and the core–shell structure of the Fe_3_O_4_@SiO_2_ nanoparticles was confirmed by a transmission electron microscope (TEM) (Figure S1, Supporting Information). A microfluidic device was constructed, whose main part was composed of two coaxially assembled cylindrical glass capillaries (Figure S2, Supporting Information). Two fluids were pumped into the device forming a coflow regime. An aqueous suspension of Fe_3_O_4_@SiO_2_ nanoparticles served as the inner phase, and an oil phase consisting of 90% (v/v) glycerol triacetate and 10% (v/v) silicon oil was the outer phase. When the two phases met at the intersection, the inner phase fluid was pinched off into droplets, and the real‐time images of such emulsification process were shown in Figure 2b. After generated from a coflow microfluidic device, the droplet templates were collected into a container filled with glycerol triacetate, which served as a water extractant to accelerate the concentrating process of the nanoparticles. During the concentrating procedures, an external vertical magnetic field was applied to induce the self‐assembly of the magnetic nanoparticles toward a highly ordered hexagonal close packing, and such periodically ordered lattices remained stable with the rapid water extraction. Meanwhile, the magnetic force in vertical directions led to stretching of the droplet interface and the droplets deformed from spherical to spindle shape with time. In addition, the directional magnetization of the magnetic nanoparticles imparted the final spindles with magnetic property for subsequent magnetic manipulations.

Under an external magnetic field, the nanoparticles in suspension would be induced to form photonic crystals (PhCs) and appear brilliant structural colors, as presented in Figure 2c and Figure S3 (Supporting Information). Accordingly, the Fe_3_O_4_@SiO_2_ nanoparticles in droplets in the extractant solution also presented respective structural colors with magnetism (Figure 2d). We acquired optical images of the droplets from side view to characterize the entire magnetism‐mediated self‐assembly process, as depicted in Figure S4 (Supporting Information). Initially, due to the higher density of glycerol triacetate (*ρ* = 1.16 g cm^−3^) than water, the droplet floated at the gas–liquid interface and appeared intrinsic pale brown color. When a vertical magnetic field was exerted, the internal nanoparticles underwent ordered assembly and the droplet revealed green structural colors. Along with the volume decreasing accompanying water extraction, the vertical magnetic force performed continuous stretching on the droplets. The droplets deformed at the long axis and finally displayed a spindle geometry. To better optimize the assembly process of the SCSPs, we analyze the two effects, namely solvent extraction and magnetic deformation, as shown in Figure 2e. The optical images of the SCSPs during the entire process were recorded with time (Figure 2f). The solvent extraction and magnetic deformation contributed to the formation of the final geometry, and the droplet confined self‐assembly process imparted the SCSPs with brilliant structural colors. It was observed that the shape of the droplets converted from spheroid to spindles with time, until complete solvent extraction that led to fully solidification of the SCSPs. The deformation process was characterized by measuring the oblateness as a function of time (Figure 2g), which was defined as

(1) $$\mathrm{Oblateness}=\left(L-D\right)/D\times 100\%$$

where *L* is the length along the long axis of the SCSP, and *D* refers to the distance along the short axis of the SCSP.

**Figure 3:**
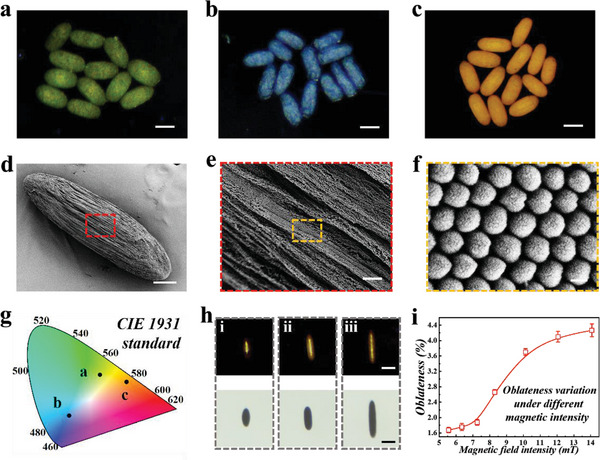
Synthesis and characterization of the SCSPs. Optical images of the SCSPs after calcination, bearing a) green, b) blue, and c) orange structural colors. d–f) SEM images of the microstructures of the SCSPs under different magnifications. g) The CIE 1931 standard chromaticity diagram of the above three SCSPs. h) The transmission and reflection images of the SCSPs fabricated under i) *B* = 5.83 mT, ii) *B* = 8.26 mT, and iii) *B* = 14.47 mT. i) The fitted curve of oblateness of the SCSPs as a function of the magnetic field intensity. Scale bars are 200 µm in (a–c), 50 µm in (d), 2 µm in (e), 100 nm in (f) and 200 µm in (h).

It was worth mentioning that the internal highly ordered lattices would not be disturbed during the assembly process, and thus the final spindle still remained corresponding structural colors. Specifically, the structural colors of the SCSPs could be indicated by their characteristic reflection spectra, whose peak position *λ* could be calculated according to the Bragg's equation for normal incidence

(2) $$\lambda =1.633dn_{\mathrm{average}}$$

where *d* is the center‐to‐center distance between neighboring Fe_3_O_4_@SiO_2_ nanoparticles, and *n*_average_ refers to the average refractive index of the SCSPs. This revealed that the structural colors of SCSPs could be tuned by altering *d*, that is, the diameter of the nanoparticles. Accordingly, we obtained three types of SCSPs with different colors. Subsequently, the spindles were calcined to further enhance their mechanical strengths; their final images were shown in **Figure** **3**a–c. We used EDS mapping to identify the chemical elements of the SCSPs after calcination, and the results demonstrated that final SCSPs were composed of Fe, Si, and O (Figure S5, Supporting Information). Besides, we used SEM to characterize the microstructures of the SCSPs. As displayed in Figure 3d–f, abundant ravines appeared at the surface of the SCSPs, which were formed by closely stacked nanoparticles. This implied that the magnetism self‐assembly was relatively faster than water extraction, and the Fe_3_O_4_@SiO_2_ nanoparticles rapidly formed highly ordered arrangements. Such microstructures imparted SCSPs with macroscopical brilliant structural colors, which were calculated into a chromaticity diagram (Figure 3g). Such hierarchical structure endowed the SCSPs with sufficient specific surface area, which served as modification sites for bioactive substances. In addition, we optimized the intensity of the external magnetic field and tuned the final oblateness of SCSPs (Figure 3h). The initial droplets were set to be same and deformed under different magnetic field intensity until final morphology was fixed. It was found that stronger magnetic field led to larger oblateness due to stronger magnetic stretching effect performed on the droplets (Figure 3i). However, too strong magnetic force would hamper the self‐assembly of the nanoparticles, hence the magnetic field intensity was set as 14.47 mT.

**Figure 4:**
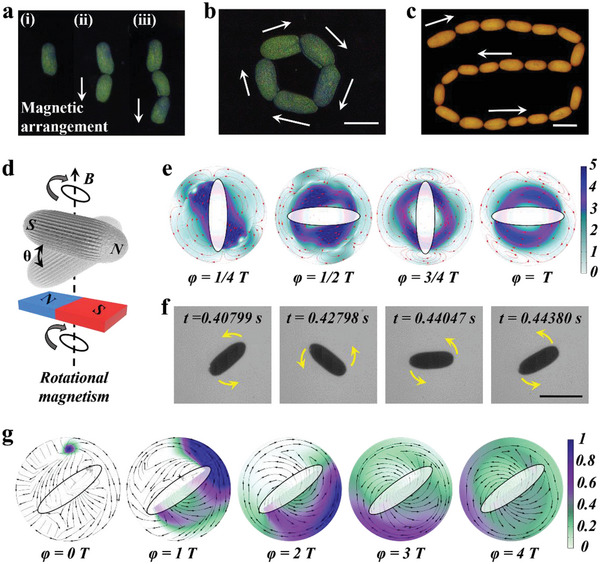
Magnetism‐based manipulation and rotary behavior of the SCSPs. a) Reflection images of green SCSPs arranged in a chain along the magnetic field direction. b,c) Reflection images of green and yellow SCSPs arranged to form various patterns. The white arrows refer to the variations of magnetic field directions. d) Schematic of the rotary motion ability of the SCSP induced by rotational magnetism. e) The flow velocity and streamline distribution around the SCSP during one rotation period. f) Time‐lapse images of a single SCSP during one spinning period at 1500 rpm under a magnetic field (*B* = 1.45 mT). g) The variations of the concentration distribution of a model substance released around the SCSP along with the rotation. Scale bars are 350 µm in (b, c, f).

We next explored the magnetic properties of the prepared SCSPs. Magnetic hysteresis loop of the SCSPs was acquired by vibrating sample magnetometer (VSM) (Figure S6, Supporting Information). The result indicated that the SCSPs are soft magnetic materials easy to be magnetized. We speculate that the Fe_3_O_4_@SiO_2_ nanoparticles initially presented random magnetic moment arrangements in droplets. When adding the constant vertical magnetic field, these nanoparticles were magnetized and arranged along the field direction. Such magnetic moment orientation kept stable throughout the whole assembly procedure, hence the final SCSPs presented fixed magnetic moment orientation along the long axis. Remarkably, the SCSPs could remain weak magnetization even when the external magnetic field was removed. Based on these properties, we realized diverse magnetic manipulations on the SCSPs. As shown in **Figure** **4**a, we used a bar magnet to arrange the SCSPs to form a chain along the field direction. After removing the magnetic field, such directional arrangement could remain for a long time based on the remanence. Similarly, by regulating the direction of the magnetic field, various arrangements bearing different patterns could be easily obtained (Figure 4b, c and Figure S7, Supporting Information).

Most intriguingly, the SCSPs showed magnetic‐controlled rotary behaviors. By integrating a rotational magnetic field, the SCSPs could spin at a tunable speed around the central axis, as schemed in Figure 4d. In a stationary flow field, integration of this spinning motion would generate turbulence and vortex, which could accelerate mixing and promote chemical reactions. To better reveal the variations of flow field caused by the rotary SCSPs, we performed numerical simulations to investigate the flow velocity and streamline distributions during magnetic‐driven spinning (Figure 4e). It was found that abundant turbulences were generated during one rotary period, and the flow velocity near the SCSP was obviously higher than other regions. Besides, we used an ultra‐high‐speed camera to capture a single SCSP spinning at a speed of 1500 rpm in a rotational magnetic field (*B* = 1.45 mT). The time‐lapse images demonstrated the stable magnetic‐induced spinning behavior during a rotary period from 0.40799 to 0.44380 s, as presented in Figure 4f and Movie S1 (Supporting Information). We also found that decreasing the distance between the SCSPs and the magnet could make SCSPs roll over (Figure S8 and Movie S2, Supporting Information), due to the action of an extra vertical magnetic force. We then explored the applications of the rotary SCSPs for accelerating mixing. We numerically simulated the concentration profile of a model molecule released around a SCSP at a certain concentration (1 mol L^−1^). It was confirmed that the molecules appeared well‐distributed after four rotary periods (Figure 4g). Furthermore, we validated that the SCSPs could be magnetically collected by using a magnet (Figure S9, Supporting Information). These excellent features impart the SCSPs with great values for controlled motion and reactions.

**Figure 5:**
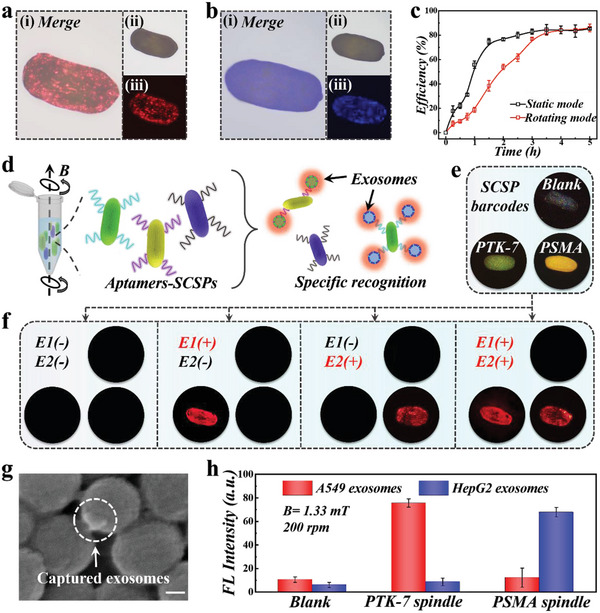
Function of the SCSPs for specific recognition of tumor cells‐derived exosomes. a) Merged, bright field and fluorescent images of a PTK‐7 aptamer‐functionalized SCSP capturing A549 cell‐derived exosomes. b) Merged, bright field and fluorescent images of a PSMA aptamer‐functionalized SCSP capturing HepG2 cell‐derived exosomes. c) The capture efficiency of target exosomes by SCSPs under rotating mode and static mode, respectively. d) Schematic illustration of encoding strategy based on SCSPs with three structural colors for detecting two kinds of tumor‐derived exosomes. e,f) The reflection and fluorescent images of a blank SCSP (blue), PTK‐7‐SCSP (green), and PSMA‐SCSP (yellow) for detection of A549 exosomes (E1) and HepG_2_ exosomes (E2). g) SEM image of exosomes captured on the surface of the SCSPs. h) Fluorescent intensity of three SCSP barcodes applied for specific exosomes detection. Scale bar is 50 nm in (g).

As mentioned above, Fe_3_O_4_ @SiO_2_ nanoparticles possessed a silica shell coating, making it easy for surface modification with bioactive substances. In addition, the large specific surface area endowed SCSPs with sufficient combining sites. With this, the SCSPs were functionalized with single‐stranded DNA (ssDNA) aptamers for specific recognition and capture of tumor cells‐derived exosomes. Herein, PTK‐7 aptamer targeting lung cancer A549 cells‐derived exosomes or PSMA aptamer targeting liver cancer HepG2 cells‐derived exosomes were functionalized on the SCSPs through simple modification procedures (Figure S10, Supporting Information, and Experimental Section), and the successful functionalization was confirmed by fluorescence imaging (Figure S11, Supporting Information). The DNA sequence of two aptamers were presented in Table S1 (Supporting Information). Next, A549 cells‐derived exosomes and HepG2 cells‐derived exosomes were extracted by standard ultracentrifugation procedures, and TEM images of the exosomes were presented in Figure S12 (Supporting Information). We labeled A549 cells‐derived exosomes with 1,1′‐dioctadecyl‐3,3,3′,3′‐tetramethylindocarbocyanine perchlorate (Dil) and HepG2 exosomes with Hochest for easier distinction. It was demonstrated that both two exosomes were captured by respective aptamer‐SCSPs with high efficiency (**Figure** **5**a,b). Besides, we used confocal fluorescent microscope to characterize the SCSPs capturing exosomes on the surface, as shown in Figure S13 (Supporting Information). The dynamic light scattering (DLS) size distribution was measured, which showed that these exosomes possessed a monodispersed size of ≈60–100 nm (Figure S14, Supporting Information).

Besides, we recorded the fluorescent intensity of the SCSPs during static detection, as shown in Figure S15a (Supporting Information). Three SCSPs were incubated with 200 µL exosomes solution, and the real‐time fluorescent intensity was acquired. We found that the fluorescent intensity reached saturation at about 3 h, which indicated that the combination sites on SCSPs were nearly fully occupied with target exosomes. Especially, we compared the capture efficiency at static mode and rotating mode respectively by measuring the fluorescent intensity at the same time points. Rotating mode was set at a speed of 150 rpm under a magnetic field (*B* = 1.38 mT). The results revealed that the capture efficiency in the rotating mode reached saturation eariler than the static mode (Figure 5c). This was mainly ascribled to the turbulence generated by the rotary SCSPs, which contributed to rapid contact and reaction between the exosomes and the SCSPs. Apart from this, we optimized the experimental parameters for exosome detection. As shown in Figure S15b (Supporting Information), the saturation fluorescence intensity displayed a positive relationship with the aptamer concentration. It was worth noting that blank SCSPs also showed weak fluorescence, probably due to unspecific recognition effect. The concentration of aptamer was set as 20 × 10^−6^ m for subsequent experiments. Also, the reaction temperature was optimized at 37 °C for subsequent experiments (Figure S16, Supporting Information).

Bearing both rotatory motion ability and the structural color feature, the SCSPs are available as intelligent barcodes for multiplex exosomes detection (Figure 5d). We prepared SCSPs of three colors (blue, green, and yellow), and modified them with specfic DNA aptamers (blank, PTK‐7, PSMA) as encoding information, respectively (Figure 5e). Similarly, A549 and HepG2 exosomes were labeled with Dil dye, and thus we could distinguish the exosomes by red fluorescence. The feasibility of multiple detection of exosomes was explored using SCSPs in the rotating mode in four solutions containing no exosomes, only A549 or HepG2 exosomes, and both two exosomes, respectively. As shown in Figure 5f, SCSPs of different structural colors only recognized corresponding target exosomes, which enabled encoded detection of multiple tumor cells‐derived exosomes. The three kinds of SCSPs could be easily differentiated by the chromaticity diagram (Figure S17, Supporting Information). SEM images of the SCSPs after detection confirmed that the exosomes were captured on the surface of the SCSPs (Figure 5g). Besides, we applied three kinds of rotary SCSPs in solution samples containing A549 or HepG2 exosomes, the specific detection could be identified through bright field and fluorescence imaging (Figure S18, Supporting Information), and the fluorescent intensity of each SCSPs also helps to distinguish the detection process (Figure 5h). Compared with other optical encoding methods, such encoding strategy possessed stability and flexibility owing to the unique structural color and rotary features of the SCSPs.

**Figure 6:**
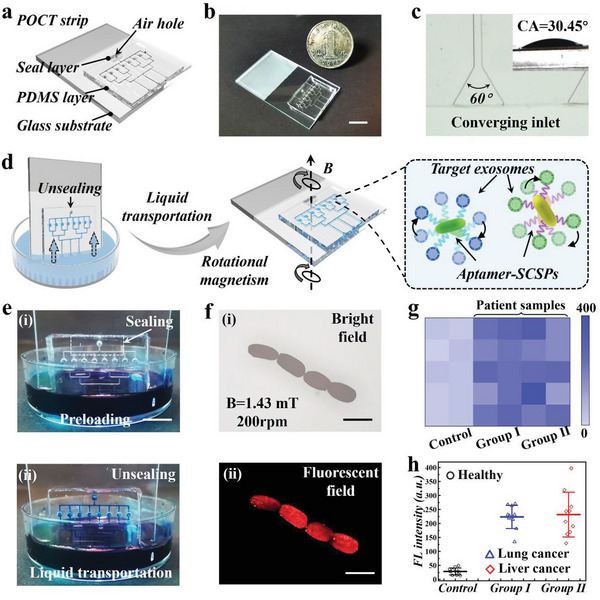
Construction of a POCT strip integrated with SCSPs for auxiliary tumor diagnosis. a) Schematic and b) photograph of the c) optical microscopic image of the converging inlet and the contact angle after hydrophilic treatment. d) Schematic diagram of the sample loading procedure of the POCT strip and the subsequent turbulence‐assistant specific exosome detection procedures. e) The strip i) at the preloading state when the air hole was sealed and ii) successful liquid transport when the hole was unsealed. The Brilliant Blue serves as the model solution to verify the liquid transportation function. f) The bright field and fluorescent images of SCSPs combining target exosomes in reaction chambers after magnetism‐induced rotation. g,h) Heat map and scatter diagram of the fluorescent intensity of SCSPs in POCT strip for detection of exosomes in the serum samples of healthy people (*n* = 10), lung cancer patients (*n* = 10), and liver cancer patients (*n* = 10). Scale bars are 1 cm in (b, e) and 350 µm in (f).

As a demonstration of practical applications, we designed a slippery POCT strip integrating the SCSPs for auxiliary clinical tumors diagnosis, before which the biocompatibility of the SCSPs was examined by coculturing with NIH‐3T3 cells (Figure S19, Supporting Information). The POCT strip was composed of three layers, including a top seal layer, a middle polydimethylsiloxane (PDMS) layer, and a bottom glass substrate, as shown in **Figure** **6**a,b. The middle PDMS layer incoporated an air hole, reaction chambers, and parallel microfluidic channels, which were composed of three converging channels and eight transport channels (Figure S20, Supporting Information). Especially, the converging inlets were designed with an angle of 60° for better liquid transporting. In addition, the slippery strip was rendered hydrophilic with a water contact angle of 30.45° (Figure 6c). For sample loading, the POCT strip embodied a facile design that realizes liquid transportation based on pressure difference and capillary force (Figure S21a, Supporting Information). As presented in Figure 6d, the seal layer initially fitted on the middle layer to close the air hole, and the POCT strip was vertically inserted in the sample solution under the liquid level. Once removing the seal layer, the liquid could be transported into the strip from the hydrophilic converging inlets to the reaction chambers owing to the pressure difference (Figure 6e and Figure S21b, Supporting Information). Then an external a rotational magnetic field (*B* = 1.43 mT, 200 rpm) was applied to induce spinning of the SCSPs and accelerate the detection of the target exosomes. It was demonstrated that the SCSPs in the reaction chambers displayed brilliant fluorescence, whose intensity was positively related to the exosome quantity (Figure 6f). We further examined the limit of detection (LOD) of two kinds of tumor cells derived exosomes. The LOD of A549 exosomes is 1.67E4 particle mL^−1^ and that of HepG2 exosomes is 1.38E4 particle mL^−1^. Finally, we applied the POCT strip for detection of target exosomes in clinical serum samples from cancer patients. Serum samples of lung cancer patients (*n* = 10), liver cancer patients (*n* = 10), and healthy people (*n* = 10) were acquired. After detection using the POCT strip, we measured the fluorescence signals of exosomes in real samples, where the control group and two patient groups represented obvious differences, as shown in Figure 6g,h.

## 3 Conclusion

In conclusion, we reported novel rotary SCSPs by magnetic‐induced self‐assembly of colloidal magnetic nanoparticles in droplets along with solvent extraction. By adjusting the experimental parameters during the fabrication process, the magnetic SCSPs bearing brilliant structural colors and spindle‐like geometry were obtained. Especially, the directional magnetic moment arrangement imparted the SCSPs with the capability of being magnetically manipulated. Besides, the abundant specific surface area enabled the SCSPs to be functionalized with DNA aptamers for specific and efficient detection of tumor cells‐derived exosomes. SCSPs with different structural colors served as encoding barcodes for detection of multiple exosomes with high sensitivity and accuracy. Moreover, the spinning SCSPs could induce turbulence in a detection fluid and thus significantly improve the capture efficiency and shorten the detection time. Based on this, we developed a portable POCT strip integrated with rotary SCSPs for auxiliary clinical tumor diagnosis via a facile hydrophilia‐driven sample loading. These features, together with the excellent biocompatibility, make the rotary SCSPs highly promising for POCT and liquid biopsy applications. Our study offered a magnetic‐induced, droplet‐confined assembly approach for the generation of anisotropic colloidal crystal particles, and demonstrated the value of such shape anisotropy for practical applications.

## 4 Experimental Section

### Materials

Ethylene glycol (EG), sodium acetate (NaAc), anhydrous ferric chloride (FeCl_3_), l‐ascorbic acid (Vitamin C), 1,1′‐dioctadecyl‐3,3,3′,3′‐tetramethylindocarbocyyanine perchlorate (Dil), and Hochest dye were purchased from Sigma‐Aldrich. Sodium hydroxide (NaOH), tetraethoxysilane (TEOS), ammonium hydroxide (NH_3_·H_2_O), glycerol triacetate, poly(4‐styrenesulfonic acid‐*co*‐maleic acid) sodium salt (PSSMA, Mw ≈ 20 000, SS:MA = 1:1), silicone oil (50cs), (3‐aminopropyl)triethoxysilane (APTES), 2‐morpholinoethanesulfonic acid (MES), N‐hydroxysuccinimide (NHS), and 1‐(3‐dimethylaminopropyl)‐3‐ethylcarbodiimide hydrochloride (EDC) were obtained from Shanghai Macklin Biochemical Co. Ltd. All DNA aptamers were synthesized by Sangon Biotech Co., Ltd. (Shanghai, China) and purified by ProStar HPLC (Varian) with a C18 column. NIH‐3T3 cells were purchased from Cell Bank of the Chinese Academy of Sciences (Shanghai, China). A549 and HepG2 cells were bought from Procell Life Science & Technology Co., Ltd. (Wuhan, China). The clinical serum samples of healthy people and patients were collected from the second Affiliated Hospital of Nanjing Medical University of China by standard venipuncture (IRB protocol number [2020]‐KY‐003). Distilled water used in all experiments was purified by a Milli‐Q Plus 185 water purification system (Millipore, Beford, MA) with the resistivity higher than 18 MΩ cm.

### Synthesis of Fe_3_O_4_@SiO_2_

First, 16 mL EG, 0.26 g FeCl_3_, 1.2 g NaAc, 0.4 g PSSMA, 4.5 mg Vitamin C, and 20–50 µL H_2_O were added into a glass vial in sequence. A magnetic stirrer was added to stir at 1500 rpm for 30 min at room temperature. Then 0.24 g NaOH was rapidly added into the solution under stirring until the solution turned transparent. The solution was transferred into a stainless‐steel autoclave to undergo the hydrothermal reaction at 190 °C for 9 h. After cooling down to room temperature, monodispersed Fe_3_O_4_ nanoparticles were obtained and were washed with 1:1 ethanol/water mixture and deionized water for three times. Finally, the nanoparticles were dispersed in deionized water. By tuning the water volume during the reaction process, Fe_3_O_4_ nanoparticles of different particle sizes were prepared. Subsequently, core–shell Fe_3_O_4_@SiO_2_ nanoparticles based on typical Stöber method were synthesized. 6 mL aqueous suspension of the Fe_3_O_4_ nanoparticles was mixed with 40 mL ethanol and 2 mL NH_3_·H_2_O. The mixture was sonicated for 10 min in ice‐bath and then transferred into a three‐necked flask. After stirring in a 50 °C water‐bath at 600 rpm for 10 min, 200 µL TEOS was slowly added drop‐by‐drop at an interval of 2 min. This time interval determined the thickness of the silica shell coated on the surface of the Fe_3_O_4_ nanoparticles. After continuous stirring for 1 h, Fe_3_O_4_@SiO_2_ nanoparticles were collected and washed with ethanol and water for three times. Under the external magnetic field, the Fe_3_O_4_@SiO_2_ nanoparticles suspensions would appear brilliant structural colors according to the particle size and magnetic intensity.

### Preparation of SCSPs

The SCSPs were prepared via magnetism self‐assembly in droplets formed in a self‐prepared coflow microfluidic device. An aqueous suspension containing monodisperse Fe_3_O_4_@SiO_2_ nanoparticles served as the inner phase. A mixture solution of 90% (v/v) glycerol triacetate and 10% (v/v) silicon oil served as the outer phase. The two fluids were separately injected into the coflow capillary microfluidic device. The flow rates of the inner and outer phase were, respectively, set as 0.2 and 2 mL h^−1^, respectively. The aqueous phase flow pinched off into droplets, and the droplets were collected in a container filled with glycerol triacetate. One magnet was placed at the bottom of the container to produce a vertical magnetic field. The distance between the magnet and the bottom container was set as 5 cm, where the magnetic intensity was measured as 10 mT. Along with the water being extracted from the droplets by the solvent extractant, the volume of droplet decreased with time. Meanwhile, the vertical magnetic field induced the droplet to deform from spheroid to spindle. After the water was totally extracted and the spindle was fully solidified, SCSPs were magnetically separated from the solvent extractant and treated with ethyl alcohol for three times. Then SCSPs were calcined at 600 °C for 4 h under nitrogen protection. To modify the amino‐DNA aptamers on the surface of the SCSPs, 5% (v/v) APTES‐ethanol was adopted to immerse and modify the SCSPs with amino groups for 12 h. Succinic anhydride was subsequently added into the solution overnight. The free carboxyl groups of succinic anhydride were activated based on EDC/NHS reaction at a weight ratio of 7:11 in an MES solution (pH = 6.0) for 3 h at 37 °C. Finally, amino‐DNA aptamers were coincubated with the SCSPs at 25 °C for 5 h, and aptamer‐SCSPs were successfully acquired.

### Culture of Cells and Extraction of Exosomes

A549, HepG2 cells, and NIH‐3T3 cells (for biocompatibility test) were all cultured with Dulbecco's modified Eagle medium (DMEM) in a humidified incubator at 37 °C with 5% CO_2_ supply. The medium was supplied with 15% (v/v) fetal bovine serum (FBS) and 1% (v/v) penicillin‐streptomycin. The supernate of the culture medium was collected every 2 d, and the supernant was stored in a 4 °C refrigerator. The supernant was first purified via filter membrane (0.22 µm) to remove residual cell fragments. Then the exosomes were centrifuged from the supernants for 1.5 h at a speed of 44 000 rpm, and such process was repeated twice. The exosomes extracted were resuspended via phosphate buffer saline (PBS, pH = 7.4), and stored at −80 °C for several days.

### Biocompatibility Test of SCSPs

The SCSPs were pretreated with ethanol (75% (v/v)) for 24 h, and immersed in sterile PBS solution under UV light irradiation overnight. Control group was NIH‐3T3 cells cultured in multiwell plate, and experimental group was the cells cultured with SCSPs. To test the biocompatibility of SCSPs, 50 µL 3‐(4,5)‐dimethylthiahiazo(‐z‐yl)‐3,5‐di‐phenytetrazoliumromide (MTT) were added into 500 µL medium, and then the cells were put back into the incubator for subsequent 4 h. The mixture was removed and 500 µL dimethylsufoxide (DMSO) were added for optical density (OD) value measurement at 490 nm. These steps were repeated for 3 d every 24 h.

### Fabrication of Tumor Diagnosis POCT Strip

The POCT strip was composed of three layers, including a top seal layer, a middle PDMS layer, and a bottom glass substrate. Tailorable coverslip was chosen as the seal layer after hydrophilic treatment by oxygen plasma. The middle layer containing microchannels and reaction chambers was fabricated by replicating the designed patterns on a mold. PDMS base was mixed with the curing agent at a weight ratio of 10:1 and the mixture was poured onto the mold. After totally removing bubbles of the mixture by using a vacuum pump, the PDMS was cured at 80 °C for 6 h. Subsequently, the PDMS was peeled off from the substrate, and the bonding surface was treated with oxygen plasma for 3 min. The bottom glass substrate was also treated hydrophilic by oxygen plasma for chip bonding. Then the SCSPs were placed into the reaction chambers by a magnet. After that, the middle PDMS layer was placed on the bottom glass substrate with a clamp in vacuum environment at 70 °C for 1 h to realize PDMS/glass chip bonding. Then an APTES/ethanol solution was injected into the strip to better improve the hydrophilicity of the converging channels and transport channels. Afterward, the top seal layer was combined on the middle layer to seal the air hole. Finally, the tumor diagnosis POCT strip was obtained.

### Exosomes Detection

The serum samples of lung cancer patients (*n* = 10), liver cancer patients (*n* = 10), and healthy people (*n* = 10) were obtained and subsequently labeled with Dil for 30 min at 37°C. The SCSPs‐integrated POCT strip was applied to transport above sample solutions into the reaction chambers. Then the strip was placed on a rotation magnetic field (1.43 mT, 200 rpm). The fluorescent intensity of the SCSPs could be calculated based on the difference of the fluorescent intensity of solutions before and after detection, which were recorded by microplate reader at 565 nm.

### Characterization

The microstructures of the SCSPs were acquired by a field emission scanning electron microscope (FESEM, Ultra Plus, Zeiss). The core–shell structure of the Fe_3_O_4_@SiO_2_ nanoparticles and configurations of exosomes were characterized by a transmission electron microscope (JEOL, JEM‐2100). Optical images of the deforming droplets and the final SCSPs were recorded by a stereoscopic microscope (Jiang Nan) equipped with a CCD camera (Olympus, DP30BW). Time‐lapse images of the rotary SCSPs under rotational magnetism were captured by an ultra‐high‐speed camera (S‐PRI F1, AOS Technologies AG, Switzerland). The fluorescent images of SCSPs capturing exosomes were obtained by a fluorescent microscope (Olympus, BX53).

## Conflict of Interest

The authors declare no conflict of interest.

## Supporting information

Supporting information

Supporting information

Supporting information

## Data Availability

The data that support the findings of this study are available from the corresponding author upon reasonable request.
