# Supplementary material for: Rotary Structural Color Spindles from Droplet Confined Magnetic Self‐Assembly
Source: Adv Sci (Weinh). 2023 Jan 17;10(8):2207270. doi: 10.1002/advs.202207270 (PMC10015863; doi:10.1002/advs.202207270)
Supplement: Supplementary file 1 — Supporting information [file ADVS-10-2207270-s003.pdf]

ADVANCED SCIENCE
Open Access

Supporting Information

for *Adv. Sci.*, DOI 10.1002/advs.202207270

Rotary Structural Color Spindles from Droplet Confined Magnetic Self-Assembly

Hanxu Chen, Shuangshuang Miao, Yuanjin Zhao, Zhiqiang Luo and Luoran Shang**

Copyright WILEY-VCH Verlag GmbH & Co. KGaA, 69469 Weinheim, Germany, 2018.

Supporting Information

Rotary structural color spindles from droplet confined magnetic self-assembly

Hanxu Chen[1], Shuangshuang Miao[1], Yuanjin Zhao[1,3], Zhiqiang Luo[1], Luoran Shang[1,2]**

Supporting Figures

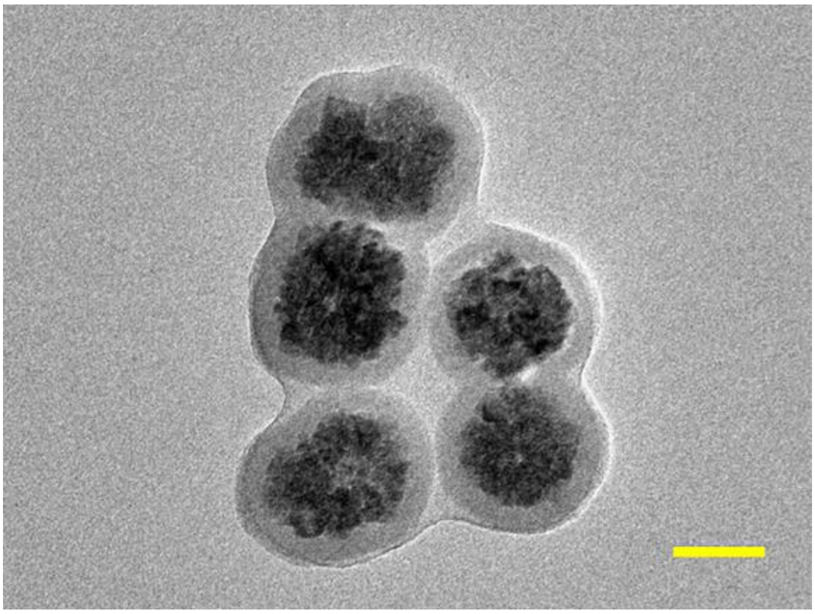

Figure S1. TEM image of the $Fe_3O_4@SiO_2$ nanoparticles. Scale bar is 100nm.

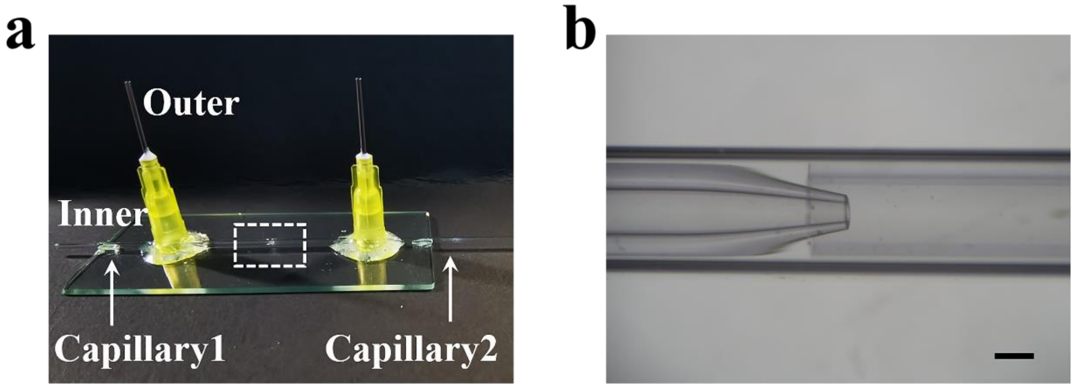

Figure S2. Optical images of the microfluidic device. (a) The architecture of the device for the fabrication of SCSPs. (b) The channel geometry of the device, with two coaxially aligned cylindrical glass capillaries assembled within a square glass capillary. Scale bar is 500μm.

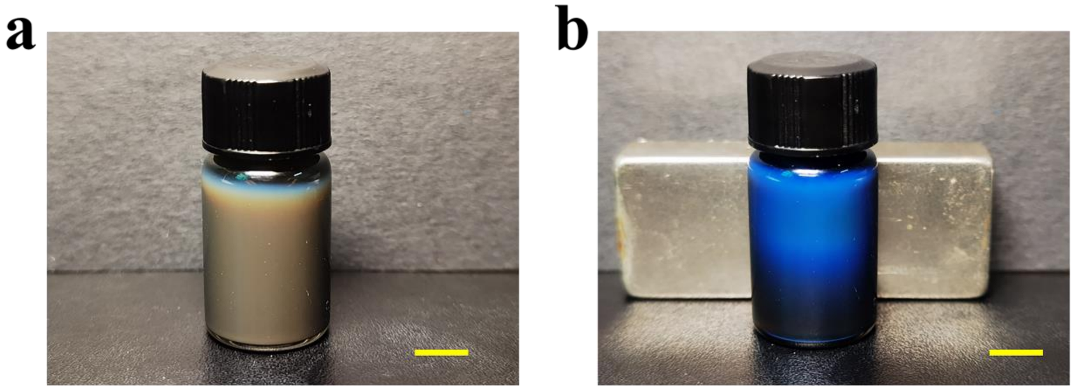

Figure S3. Reflection images of the aqueous suspension of the $Fe_3O_4@SiO_2$ nanoparticles (a) without and with (b) external magnetism. Scale bar is 7.5mm.

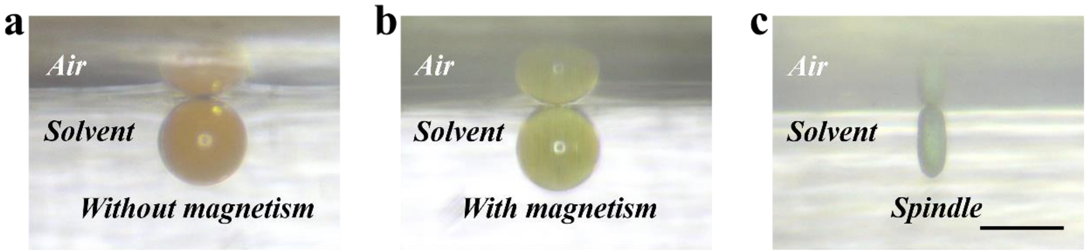

Figure S4. Reflection images of the droplet deformation process from side view: (a) the initial droplet at the air-solvent interface without magnetism; (b) magnetism-induced self-assembly and structural color appearance; (c) totally solidified SCSPs after water extraction. Scale bar is 500μm.

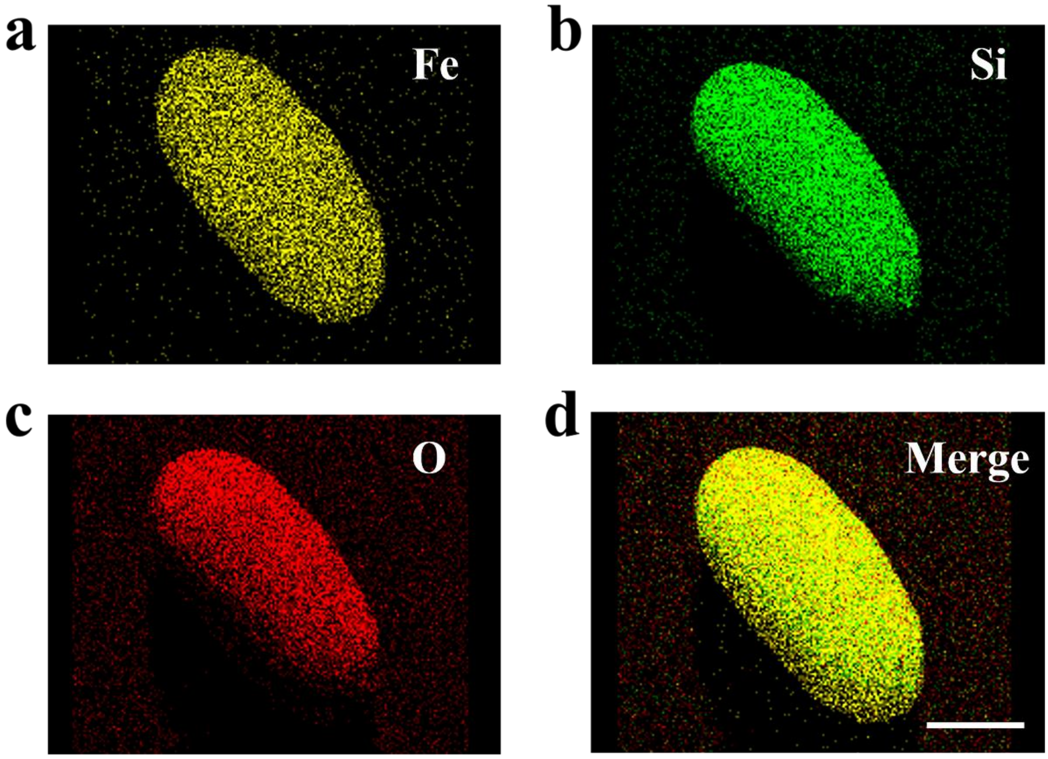

Figure S5. The element mapping of the SCSPs calcination. The SCSPs were primarily composed of Fe, Si, and O, as represented in (a), (b), and (c), respectively. (d) is the merged image. Scale bar is 175μm.

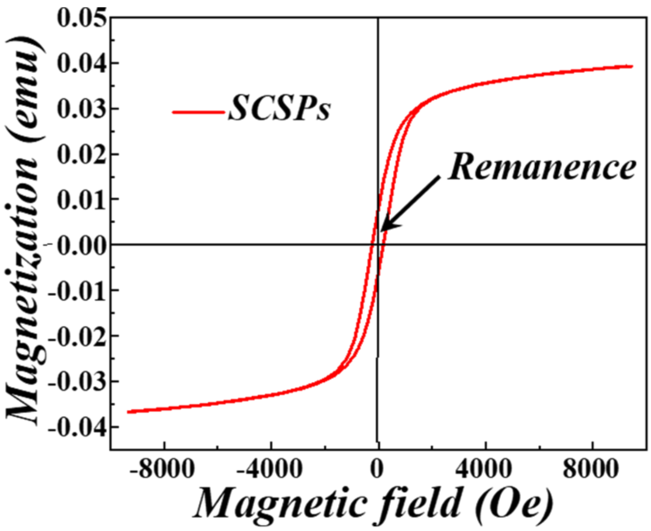

Figure S6. Magnetic hysteresis loop of the SCSPs.

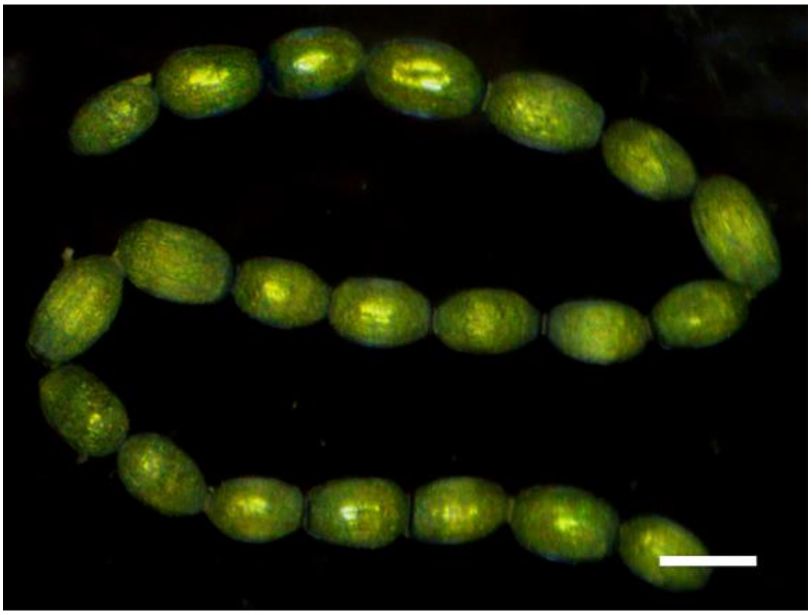

Figure S7. Magnetic arrangement of green SCSPs. Scale bar is 300μm.

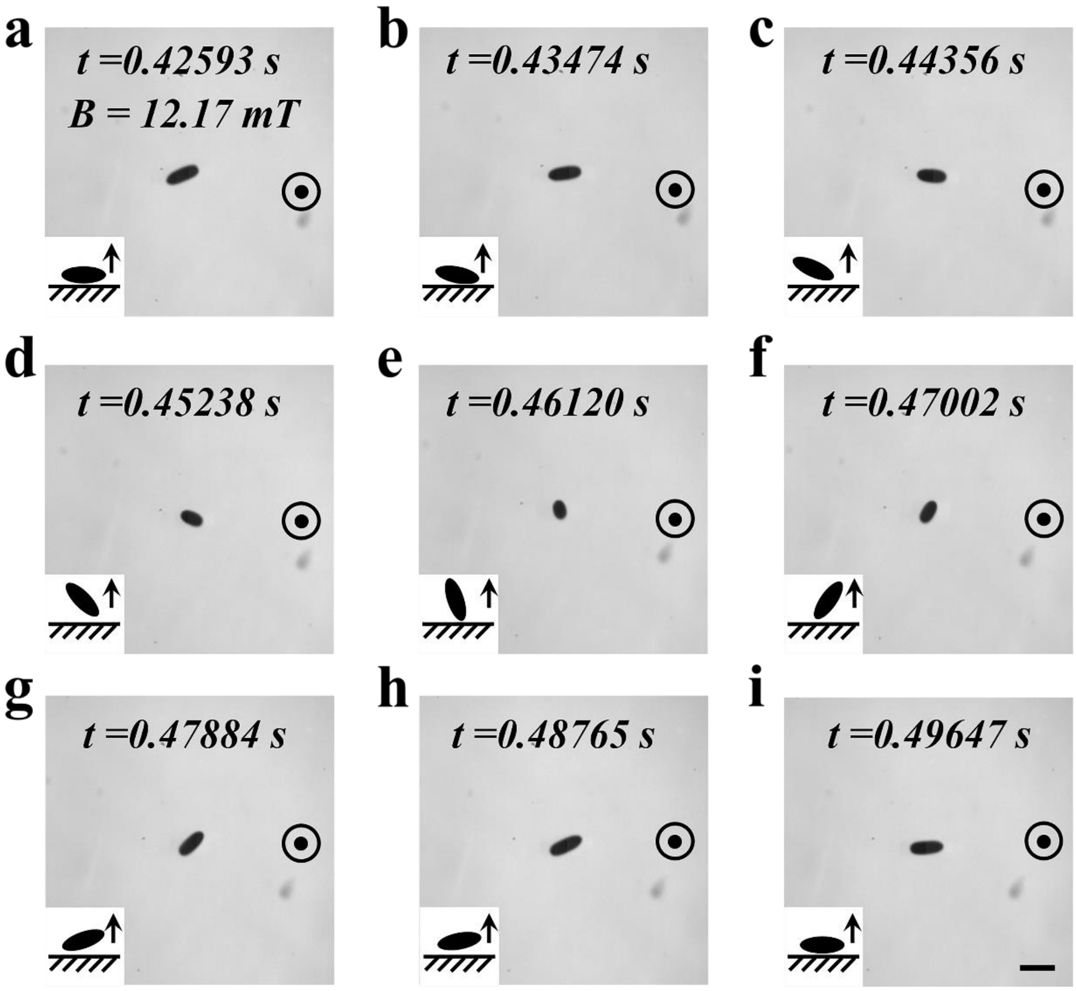

Figure S8. Optical images of the SCSPs rolling under the magnetic field (B= 12.17 mT). (a-i) Time-lapse images of a single SCSP during one rolling period. Scale bar is 300μm.

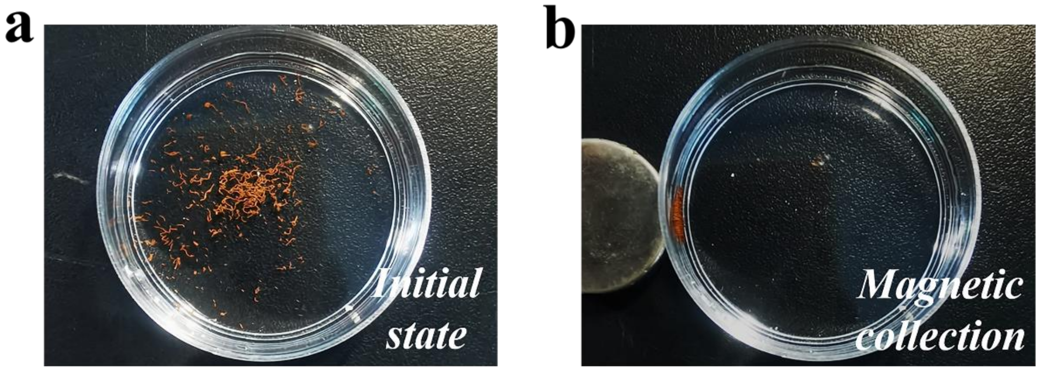

Figure S9. The reflection images of (a) randomly distributed SCSPs and (b) that after magnetic collection.

Aptamer	*Sequence (5'-3')*
PTK-7 ***(MW=15885.11)***	ATCTAACTGCTGCGCCGCCGGGAAAATACTGTACGGTTAGA-T(10)-NH_2
PSMA ***(MW=17730.24)***	GCGTTTTCGCTTTTGCGTTTTGGGTCATCTGCTTACGATAGCAATGCT-T(10)-NH_2

Table S1. The DNA sequence of PTK-7 aptamer and PSMA aptamer.

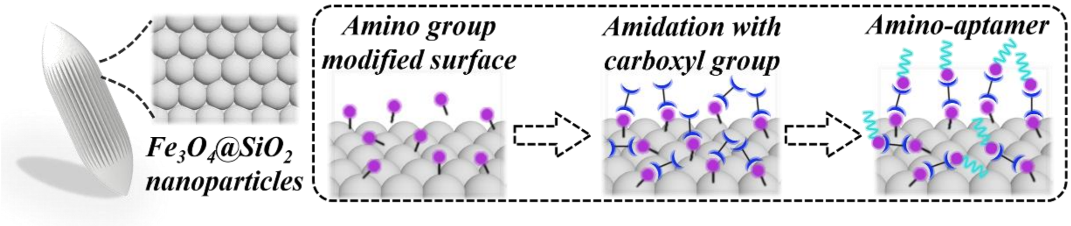

Figure S10. Schematic of the modification of DNA aptamers on the surface of SCSPs.

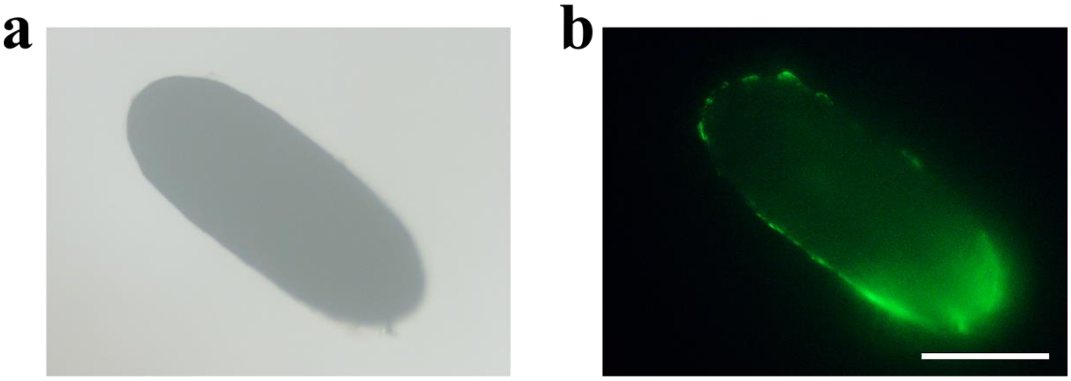

Figure S11. (a) The bright field and (b) fluorescent images of SCSPs modified with FITC-modified DNA aptamers. Scale bar is 175μm.

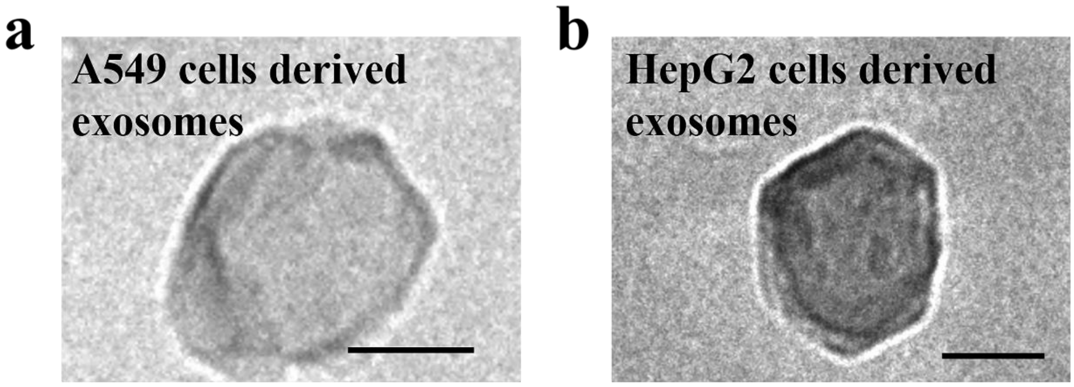

Figure S12. TEM images of (a) A549 cells-derived exosomes and (b) HepG2 cells derived exosomes. Scale bars are 50nm.

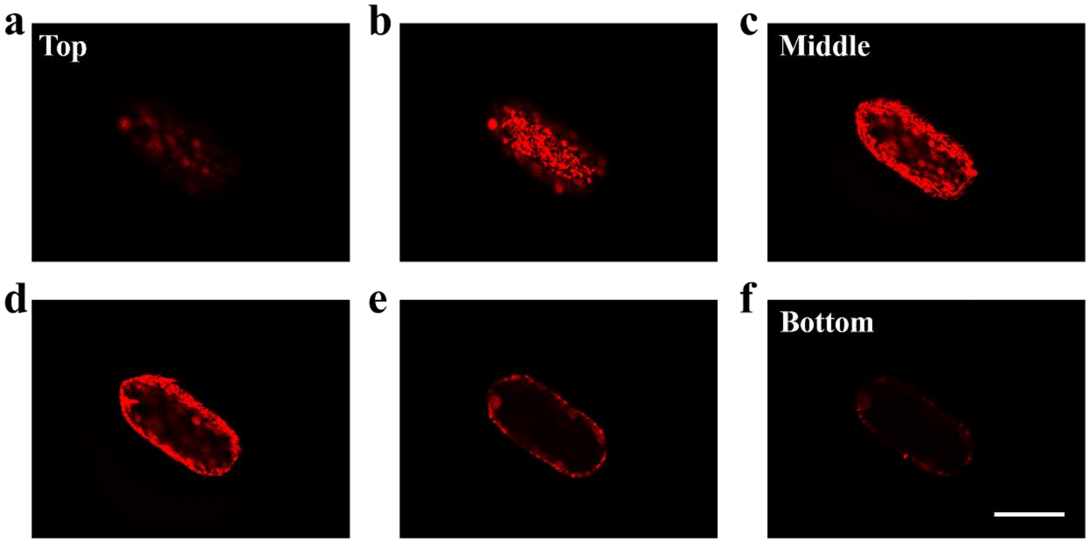

Figure S13. Confocal fluorescent images of A549 exosomes captured on the surface of PTK-7-SCSP. (a-f) Z-stack images from top to middle to bottom. Scale bar is 175μm.

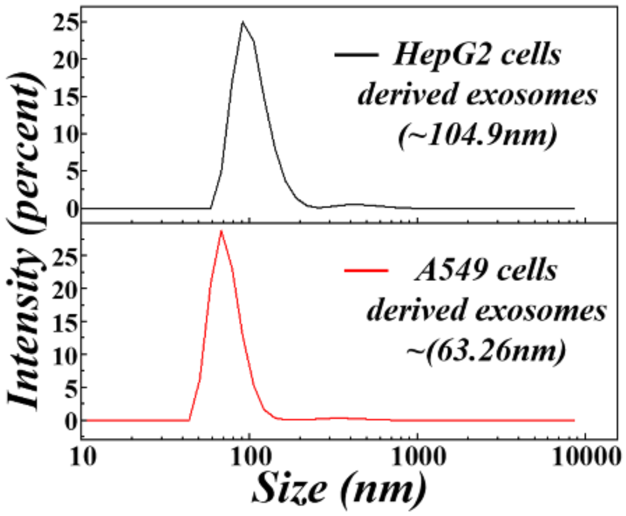

Figure S14. DLS spectrum of two kinds of exosomes.

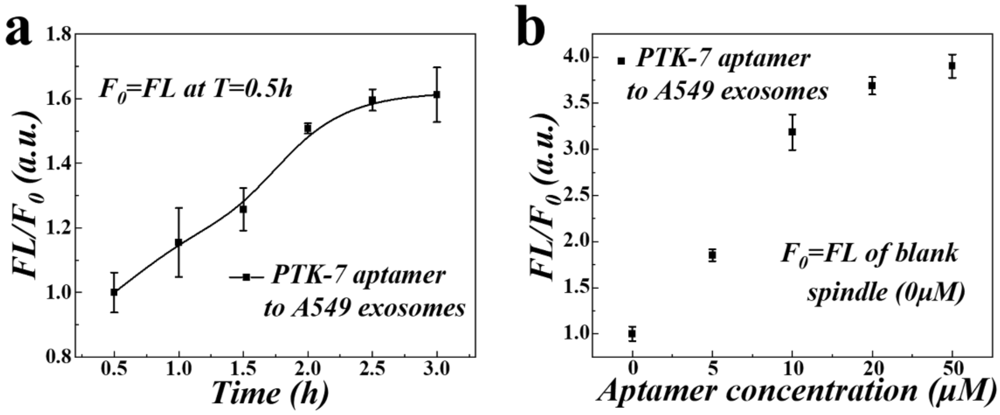

Figure S15. (a) Plot of the fluorescent intensity of the aptamer-functionalized SCSPs reacting with A549 cell-derived exosomes with time. (b) The realtionship between fluorescent intensity of exosomes-capturing SCSPs and the aptamer concentration during the modification process.

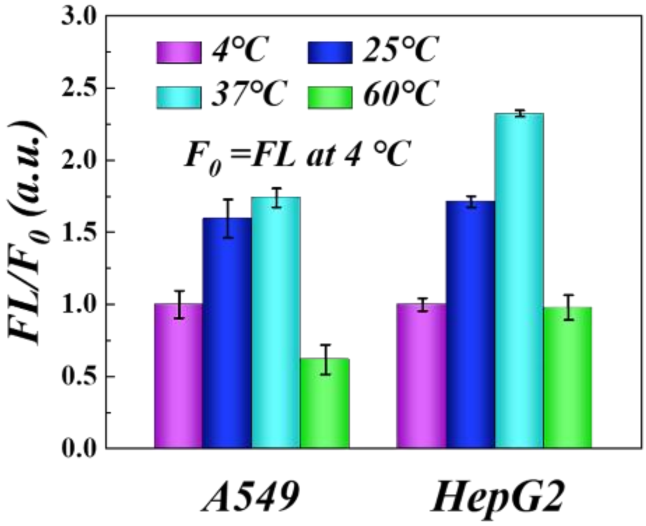

Figure S16. The fluorescent intensity of SCSPs functionalized with different aptamers capturing corresponding exosomes under four temperatures (4℃, 25℃, 37℃, and 60℃).

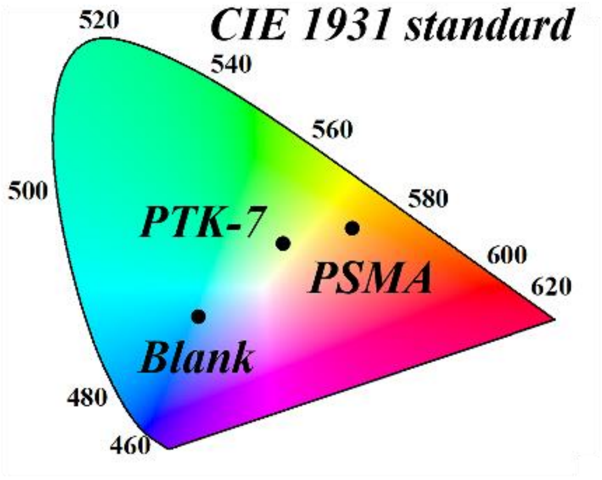

Figure S17. The CIE 1931 standard chromaticity diagram of three encoded SCSP barcodes modified with blank, PTK-7 and PSMA aptamers, respectively.

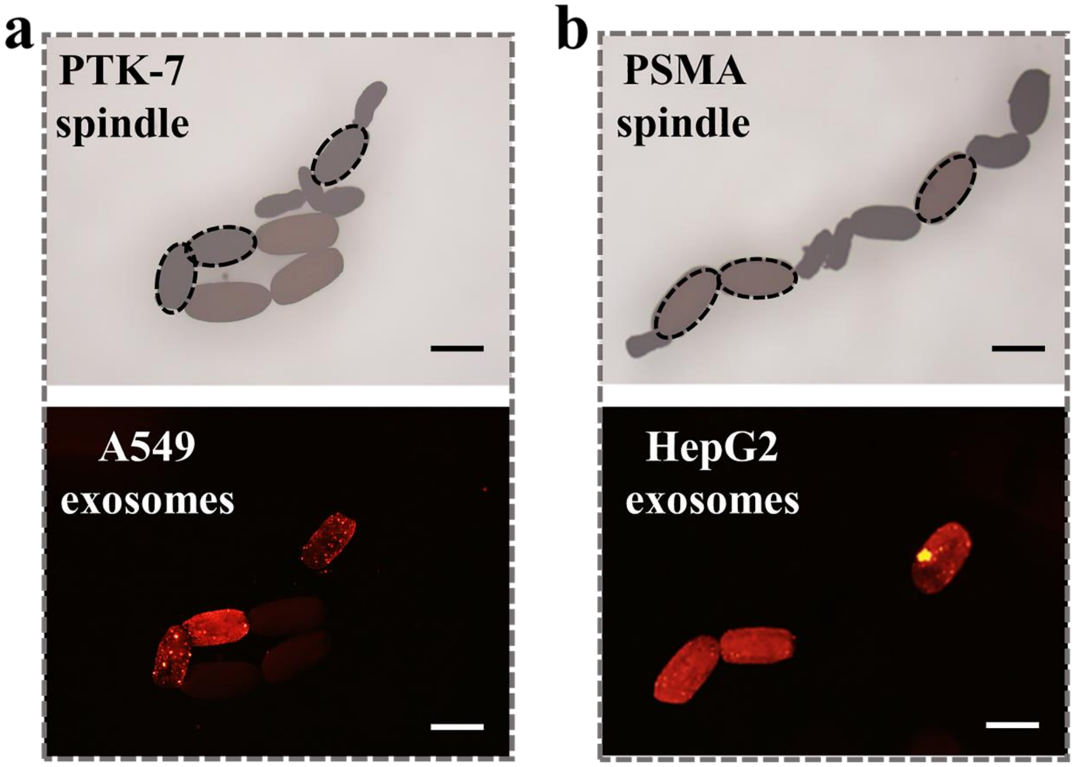

Figure S18. The bright field and fluorescent images of three kinds of SCSPs capturing Dil-labelled exosomes. (a) PTK-7 aptamer-modified SCSPs capturing A549 exosomes. (b) PSMA aptamer-modified SCSPs capturing HepG2 exosomes. The dotted lines refer to PTK-7 SCSPs in (a) and PSMA SCSPs in (b), respectively. Scale bars are 300μm.

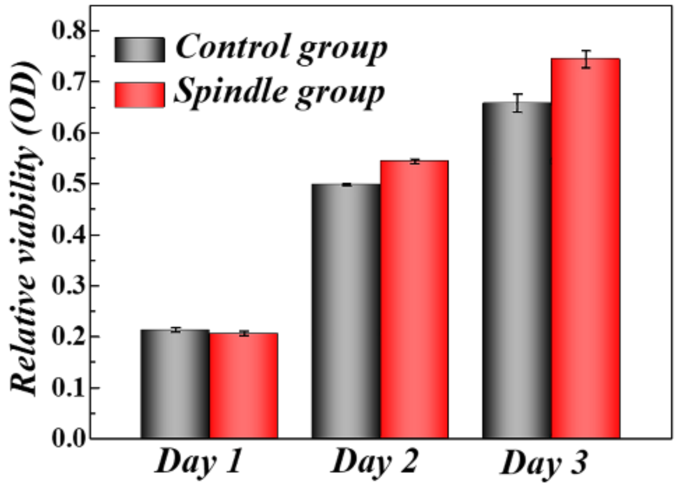

Figure S19. Evaluation of the biocompatibility of the SCSPs. Control group was NIH-3T3 cells cultured in multiwell plate, and Spindle group was cells cultured with SCSPs.

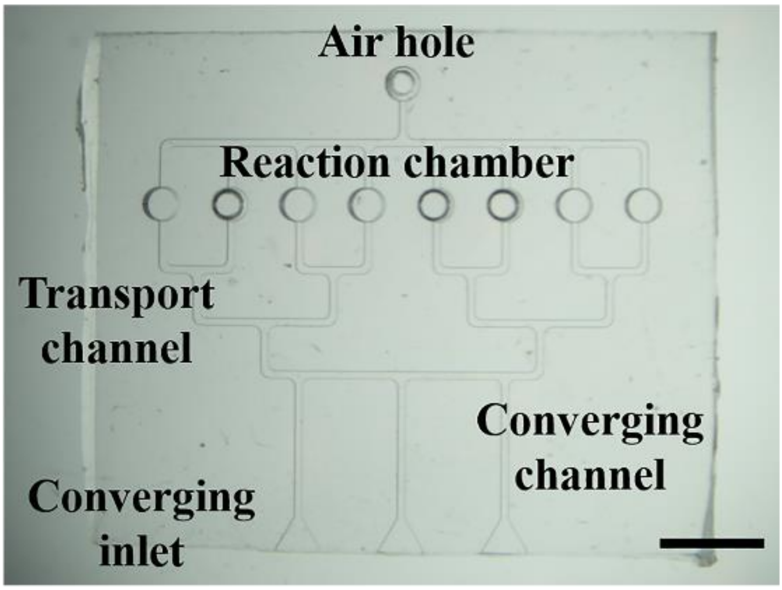

Figure S20. The optical image of the middle PDMS layer containing microchannels and reaction chambers. Scale bar is 5mm.

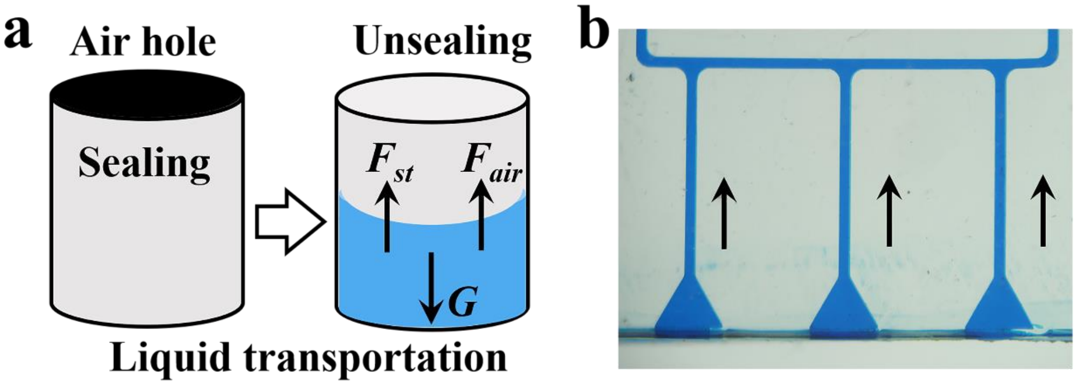

Figure S21. (a) The mechanism of liquid transportation based on the pressure difference and surface tension. F_{st} refers to the surface tension. F_{air} is the force caused by pressure difference. (b) Optical image showing the liquid transportation into the converging inlets and converging channels.

Movie S1. The video of a spinning SCSP at the speed of 1500rpm.

Movie S2. The video of a rolling SCSP at the speed of 1500rpm.
